# Hepatoprotective effect of nicorandil against acetaminophen-induced oxidative stress and hepatotoxicity in mice via modulating NO synthesis

**DOI:** 10.1007/s11356-022-23139-w

**Published:** 2022-09-23

**Authors:** Dalia H. El-Kashef, Maha H. Sharawy

**Affiliations:** Department of Pharmacology and Toxicology, Faculty of Pharmacy, Mansoura University, Mansoura, 35516 Egypt

**Keywords:** Acetaminophen, Nicorandil, Endothelial nitric oxide synthase (eNOS), Inducible nitric oxide synthase (iNOS), Nuclear factor kappa-B (NF-κB)

## Abstract

Acetaminophen (APAP) overdose can produce hepatotoxicity and consequently liver damage. This study investigated the hepatoprotective impacts of nicorandil on hepatic damage induced by APAP. Nicorandil was administered orally (100 mg/kg) for seven days before APAP challenge (500 mg/kg, ip). Pretreatment with nicorandil reduced serum levels of aminotransferases, bilirubin, GGT and LDH, and increased serum level of albumin. Moreover, nicorandil inhibited the increase in liver MDA levels and reversed the decline in GSH content and SOD activity. Besides, it notably alleviated APAP-induced necrosis observed in histopathological findings. Additionally, nicorandil alleviated APAP-induced NO overproduction and iNOS expression; however, the protein expression of eNOS was significantly increased. Moreover, nicorandil markedly reduced hepatic TNF-α and NF-κB levels, in addition to decreasing the protein expression of MPO in hepatic tissues. Furthermore, flow cytometry (annexin V-FITC/PI) displayed a significant decline in late apoptotic and necrotic cells, and an increase in viable cells in nicorandil group. Also, nicorandil caused a significant boost in hepatic antiapoptotic marker bcl-2 level. The presented data proposed that the protective effect of nicorandil might be attributed to its antioxidant, its impact on NO homeostasis, and its anti-inflammatory properties. Therefore, nicorandil may be a promising candidate for protection from liver injury induced by APAP.

**Graphical abstract:**

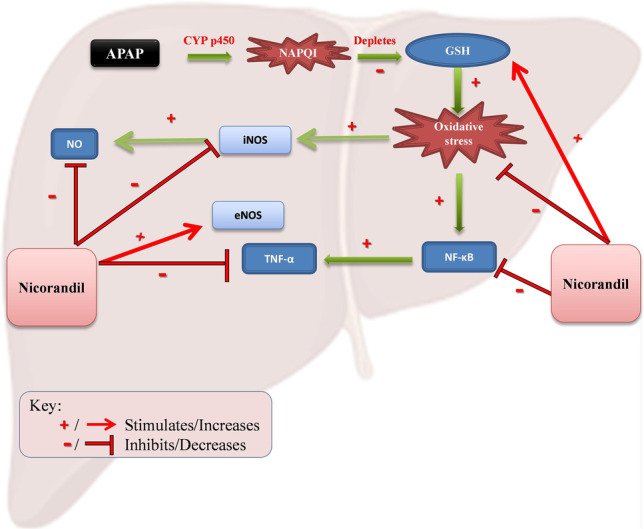

## Introduction

Despite the fact that liver is the chief organ responsible for detoxification, exposure to some drugs, infections by bacteria or viruses, environmental xenobiotics, and anticancer agents may cause liver harm and result in many liver diseases (Stephens et al. 2014). Other than any organ, the liver is more susceptible to injury due to its vital role in metabolism and its capability of concentration and biotransformation of xenobiotics (Kumar et al. 2015).

Acetaminophen (APAP), also called paracetamol, is an antipyretic and analgesic that is commonly used for children and adults in treatment of headache, fever, and different types of pain (Adam et al. 2016). Although APAP is available without a prescription, and is considered safe in therapeutic doses, overdose can result in hepatotoxicity which is fatal in both humans and experimental animals (McGill et al. 2012). APAP toxicity is a grave issue; APAP intoxication is ranked second in the world’s most common causes of liver transplantation (Agrawal and Khazaeni 2022).

Oxidative stress is an important feature in APAP model that aggravates hepatic damage. After oral administration of APAP, it is metabolized via CYP450 and produces a hepatotoxic reactive metabolite N-acetyl-p benzoquinoneimine (NAPQI) (Bromer and Black 2003). NAPQI is detoxified by reacting with glutathione (GSH) which is consumed consequently. This reaction produced alterations in mitochondrial permeability transition, followed by formation of superoxide anion radical which reacts with nitric oxide radical to produce peroxynitrite (ONOO¯) which in turn results in nitration of tyrosine protein. Additionally, peroxynitrite can decompose to hydroxyl radical which affects cell membrane integrity and triggers generation of lipid peroxides (Du et al. 2016). Accordingly, impaired mitochondrial respiration and oxidative stress are elicited, which are accompanied by colossal hepatocyte cell death, apoptosis, and induction of massive inflammatory response. Actually, elevated concentrations of pro-inflammatory cytokines, infiltrating neutrophils, and monocytes in either serum or liver are usually detected in patients with hepatic failure (Coen 2015)**.** High percentage of patients who are exposed to liver toxicity develop elevation in aspartate amino transferase (AST) and alanine amino transferase (ALT) serum levels within 24 h of ingestion (Nagi and Mansour 2000).

Nicorandil, a nitrated nicotinamide ester, is used in prevention and management of ischemic heart diseases in experimental models (Imagawa et al. 1998) and humans (Zhao et al. 2014). The ascribed pharmacological actions were due to nitric oxide (NO) donor activity and the elevation in K^+^ conductance in cardiac muscles (Taira 1987). Additionally, previous studies reported the ability of nicorandil to restore the balance between inducible nitric oxide synthase (iNOS) and endothelial nitric oxide synthase (eNOS) in models of folic acid-induced nephrotoxicity and bleomycin-induced lung fibrosis (Ezzat et al. 2021; Kseibati et al. 2020). Previous studies reported that nicorandil scavenges free radicals in STZ diabetic model (Mano et al. 2000). Moreover, it confers protection against apoptosis prompted by oxidative strain in cardiac muscles (Nagata et al. 2003) and enhances the mitochondrial dysfunction in rats with heart failure (Ahmed and El-Maraghy 2013). Lastly, it has been reported that nicorandil could ameliorate high fat diet-induced hepatic steatosis (Elshazly 2015).

This study was designated to investigate the hepatoprotective impacts of nicorandil on liver damage induced by APAP in mice and to outline the mechanisms implied, focusing on the role of nitric oxide.

## Materials and methods

### Chemicals

Perfalgan® infusion solution (Bristol-Myers Squibb, Australia) was used as a pharmaceutical source of APAP, while nicorandil was purchased as tablets (Adancor®, Merck, Egypt) and was suspended in carboxymethyl cellulose (CMC) (0.5%). All chemicals were purchased from reliable sources and were of highest analytical grade.

### Animals

Male Swiss albino mice weighing (28 g ± 2) were obtained from MERC, Mansoura University, Egypt. Mice were housed 5/cage and were retained at constant settings throughout the experiment and at room temperature 25 ºC ± 2 in a 12-h light–dark cycle. Water as well as regular diet were offered ad libitum. The study was carried out in accordance to the guidelines of Faculty of Pharmacy Ethical Committee guidelines (approval number: 2017–94/2018–16).

### Experimental design

Forty animals were randomly chosen and divided into four groups, *n* = 10.

- Group (1): Control group, mice received only CMC (0.5%, po).
- Group (2): APAP group, mice were injected with APAP once (500 mg/kg, ip) (Lim et al. 2010).
- Group (3): Nicorandil group, mice received nicorandil alone (100 mg/kg, po) for seven days; the dose was selected based on previously reported studies (El-Kashef 2018a; Matsui et al. 2015).
- Group (4): APAP/Nicorandil group, mice received nicorandil (100 mg/kg, po) for seven days and then injected with APAP (500 mg/kg, ip) on the seventh day.

### Samples collection

Twenty-four hours after APAP injection, animals were anesthetized and blood samples were withdrawn via retro-orbital sinus, centrifuged, and kept for the estimation of liver function biomarkers. Livers from all groups were rinsed in ice-cold normal saline. One part was homogenized in (10% w/v) 20 mM Tris–HCl (containing 1 mM EDTA, pH 7.4) to be used for the estimation of oxidative, inflammatory, and antiapoptotic biomarkers. The second part was used for flow cytometry assay and the last part was immersed in buffered formalin solution for 24 h and embedded in paraffin, then sliced using a microtome, for histopathological examination.

### Assessment of liver function

In serum samples, AST (Cat no. 1001160), ALT (Cat no. 1001170) albumin (Cat no. 1001020), alkaline phosphatase (ALP, Cat no. 1001130), total bilirubin (Cat no. 1001046), and gamma-glutamyl transferase (GGT, Cat no. 1001185) were assessed by standard spectrophotometric procedures using commercial kits (SPINREACT, Spain).

### Measurement of oxidative stress markers

In tris-liver homogenates, the oxidative stress biomarkers were estimated. Malondialdehyde (MDA) was assayed following the protocol described by Gerard-Monnier et al., (Gerard-Monnier et al. 1998) where N-methyl-2-phenylindole reacts with MDA in acidic pH to give a stable dye at 586 nm. GSH was determined by the reduction reaction between the thiol group of GSH and 5,5'-dithiobis(2-nitrobenzoic acid) (DTNB) to produce a yellow product that is measured spectrophotometrically (Moron et al. 1979). SOD activity was estimated in accordance to Marklund et al., (Marklund 1985). SOD activity was evaluated by calculating SOD-induced inhibition of pyrogallol autoxidation. The rise in absorbance rate at 420 nm was monitored. One unit of enzyme activity was elucidated as 50% inhibition of pyrogallol autooxidation under the assay conditions.

### Measurement of total NO concentration

Total hepatic NO was measured in liver homogenate. Nitrates were reduced by vanadium (III) chloride (VCl_3_); total nitrites were estimated using Griess reagent forming a chromophore that can be measured spectrophotometrically at 540 nm (Miranda et al. 2001).

### Measurement of lactate dehydrogenase

Serum lactate dehydrogenase (LDH) was assayed using an assay kit (Human Gesellschaft für Biochemica und Diagnstca mbH, Germany, Cat no. 12214) following the instruction manuals.

### Determination of inflammatory markers in liver homogenates

Hepatic levels of nuclear factor-kappa B (NF-κB) (Cusabio, USA, Cat no. CSB-E08789m) and tumor necrosis factor-alpha (TNF-α) (Assaypro, USA, Cat no. EMT2010-1) were measured using quantitative sandwich ELISA technique. The optical density was recorded at 450 nm.

### Histochemical determination of myeloperoxidase in liver sections

Myeloperoxidase (MPO) was estimated by histochemical method, where 3,3’ –diamino benzidine (DAB) and hydrogen peroxide were used for determination of MPO according to the manufacturer’s guidelines (Biodiagnostic Co., Giza, Egypt, Cat no. MP2611). The degree of MPO staining intensity was scored as negative, weak, moderate, and strong, each of these ordinal ranks was assigned a number from 0 to 3, respectively (Sharawy et al. 2018).

### Estimation of antiapoptotic marker bcl-2 in liver homogenates

The antiapoptotic marker B-Cell Leukemia-2 **(**bcl-2) (MyBioSource, USA, Cat no. MBS2881897) was estimated using quantitative sandwich ELISA technique. The microplate reader was set to 450 nm.

### Histopathological examinations and immunohistochemistry staining of inducible nitric oxide synthase and endothelial nitric oxide synthase

Liver sections were stained with H&E, examined microscopically and scored for necrosis by a pathologist. Lesions in 10 fields were selected haphazardly from each slide for each rat and averaged. The lesions were scored in a blinded way (score scale: 0 = normal; 1 ≤ 25%; 2 = 26–50%; 3 = 50–75%; 4 = 75–100%) (Khafaga et al. 2021, 2019).

For inducible nitric oxide synthase (iNOS) and endothelial nitric oxide synthase (eNOS) immunohistochemical staining, the serial sections were deparaffinized, hydrated, and immersed in an antigen retrieval (EDTA solution, PH 8). The sections were subsequently treated with 0.3% hydrogen peroxide and protein block; afterwards, they were incubated with rabbit polyclonal of anti-iNOS antibody (Cat no. ab15323, Abcam, UK; 1:200 dilution) or eNOS polyclonal antibody (Cat no. PA3-031A, Thermo Fischer Scientific, UK; 1:250 dilution). The slides were three times rinsed with PBS, incubated with anti-rabbit IgG secondary antibodies (EnVision + System HRP; Dako) at room temperature for 30 min. The slides were then visualized with di-aminobenzidine kits (Liquid DAB + Substrate Chromogen System; Dako), and lastly counterstained with Mayer’s hematoxylin. As a negative control technique, the primary antibody was substituted by normal mouse serum. The labelling index of iNOS and eNOS was expressed as the average percentage of positive area expression in about 5 to 8 high power fields. Assessment of immunostaining was carried out by determining the percentage of positive areas using image J analysis software. Briefly, the image is converted to grey scale and on threshold basis the ratio of positive area to total area was determined. The percentage was calculated as following: positive areas divided by total area then multiply by 100 (positive area / total area × 100) (El-Kashef 2018b). (El-Far et al. 2020).

### Flow cytometry for assessment of apoptosis and necrosis

The DNA-binding dye propidium iodide (PI) and fluorescent tagged annexin V were used to assess necrosis and the degree of apoptosis respectively. Assay was done using (Annexin V-FITC_FL1_/ PI-PE_FL2_ (Cat no. 51-65874X, Cat no. 51-66211E, respectively), BD Pharmingen™, BD Biosciences, USA) according to the enclosed manual guidelines.

### Statistical analyses

Data is expressed as mean ± standard error of the mean (S.E.M.), and *n* = 10. Different groups were compared using one-way analysis of variance (ANOVA) and Tukey–Kramer test for multiple comparisons. Differences were considered significant at *p* < 0.05. Statistical analysis was performed by GraphPad Prism software version 5 for windows (GraphPad Software Inc., San Diego, California, USA).

## Results

### Effect of APAP and/or nicorandil on liver functions

APAP induced a marked elevation in serum ALT, AST, ALP, bilirubin, and GGT (12.8, 2.5, 2.1, 4.1, and 2.5-fold change, respectively), concomitant with a profound decrease in serum levels of albumin by 12% when compared to control mice. Nicorandil significantly reduced APAP-induced alterations in serum biochemical parameters compared to APAP-treated mice by 93.7%, 63.3%, 60.1%, 52%, and 52.3%, respectively (Table 1). Whereas, nicorandil alone treated mice showed no alteration in liver function biomarkers compared to the normal mice as shown in the table, so further results regarding nicorandil alone group were dismissed for simplification.

**Table 1:** Effect of acetaminophen (500 mg/kg) and nicorandil (100 mg/kg) on liver functions

	ALT (U/L)	AST (U/L)	ALP (U/L)	Bilirubin (mg/dl)	GGT (U/L)	Albumin (g/dl)
Control	86.4 ± 4.99	363.0 ± 22.22	115.2 ± 12	0.12 ± 0.01	1.20 ± 0.18	5.0 ± 0.05
APAP	1114.4 ± 107.85^*^	940.0 ± 109.67^*^	243.55 ± 39.20^*^	0.50 ± 0.04^*^	3.02 ± 0.31^*^	4.40 ± 0.13^*^
APAP/ Nicorandil	70.0 ± 4.80^#^	344.8 ± 28.60^#^	97.0 ± 7.11^#^	0.24 ± 0.01^*#^	1.44 ± 0.26^#^	4.50 ± 0.11^#^
Nicorandil	68.2 ± 6.9^#^	329.2 ± 43.3^#^	100.0 ± 2.49^#^	0.14 ± 0.01^#^	1.10 ± 0.11^#^	5.15 ± 0.06^#$^

Mice were pretreated with nicorandil (100 mg/kg) for seven days then intoxicated with a single injection of APAP (500 mg/kg)

Data expressed as means ± SEM (*n* = 10)

^*, #^ denotes significant difference at *p* < 0.05 as compared to control group and APAP-treated group respectively (one-way ANOVA and Tukey–Kramer multiple comparisons test)

*ALP*, alkaline phosphatase (ALP); *ALT*, alanine aminotransferase; *APAP*, acetaminophen; *AST*, aspartate aminotransferase; *GGT*, gamma glutamyl transferase; *SEM* standard error of mean

### Effect of APAP and nicorandil on total hepatic NO content

**Fig. 1:**
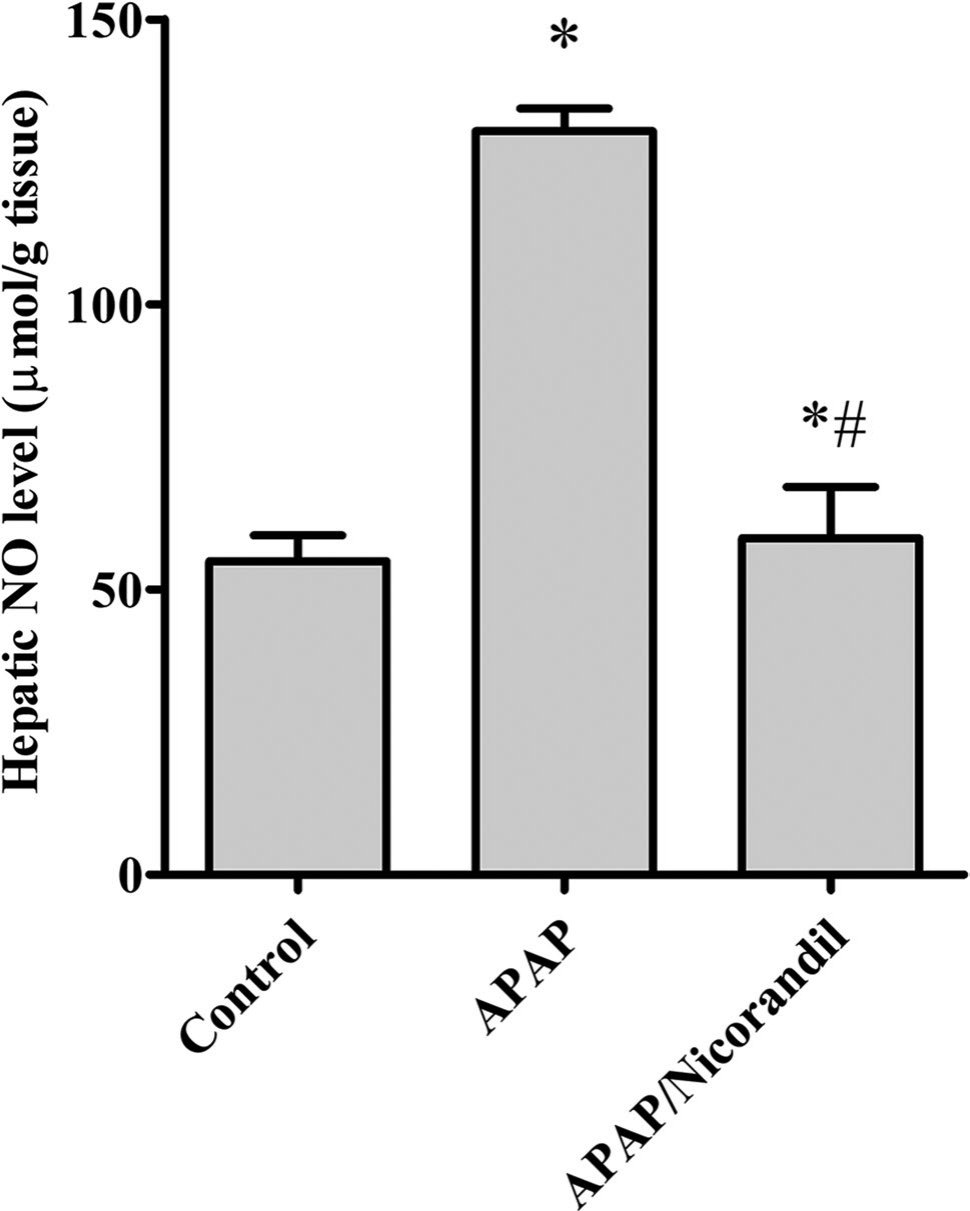
Effect of acetaminophen and nicorandil on hepatic NO content in mice. Mice were pretreated with nicorandil (100 mg/kg) for seven days then intoxicated with a single injection of APAP (500 mg/kg). ^*^, ^#^ significantly different from control and APAP-treated groups respectively (*p* < 0.05) using one-way ANOVA followed by the Tukey–Kramer multiple comparisons test. APAP, acetaminophen; NO, nitric oxide

APAP produced a significant elevation in hepatic NO content (130.3 ± 4.05) compared to the normal group (54.9 ± 4.4). Nicorandil effectively restored NO (59 ± 5.1) to the normal levels (Fig. 1).

### Effect of APAP and nicorandil on serum LDH levels

**Fig. 2:**
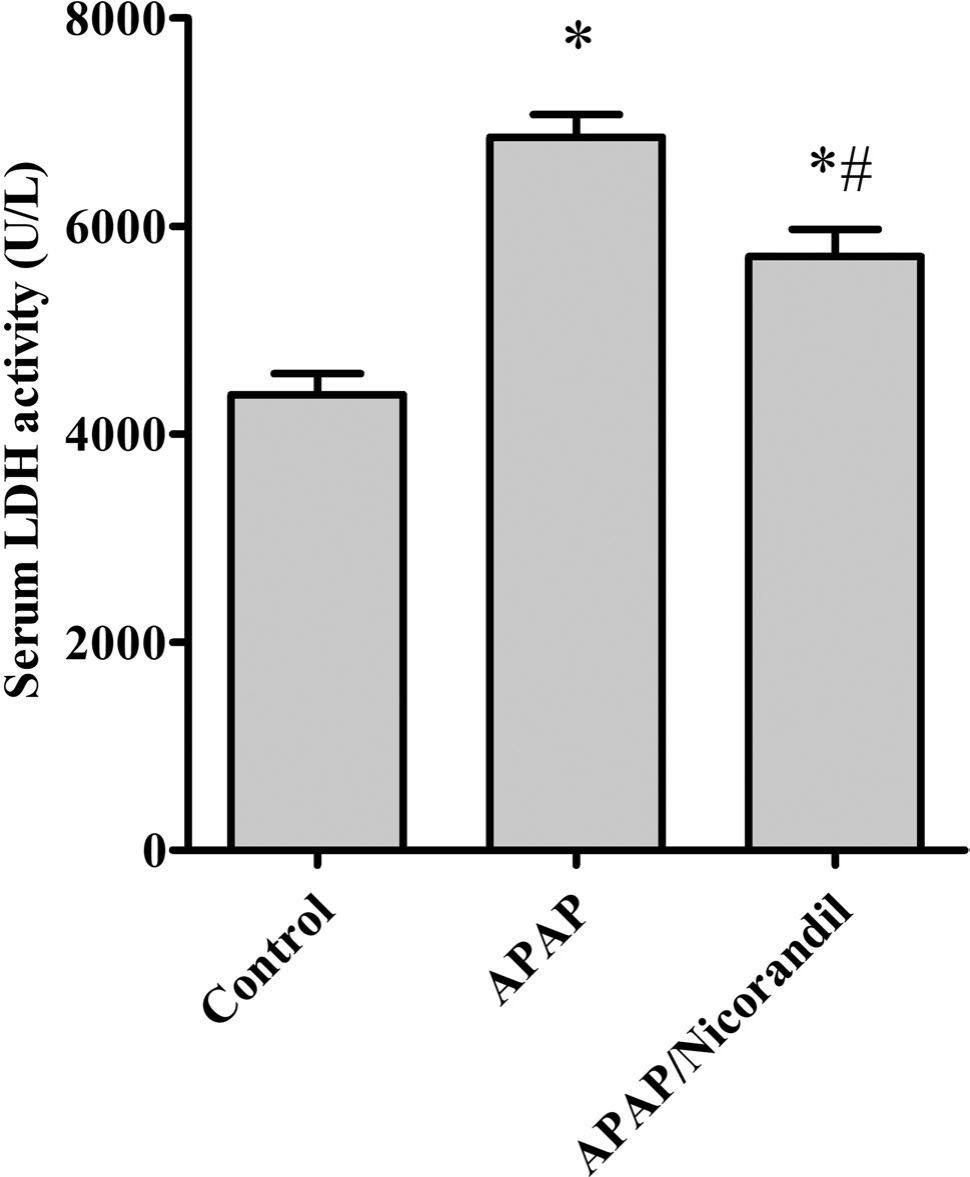
Effect of acetaminophen and nicorandil on serum LDH level in mice. Mice were pretreated with nicorandil (100 mg/kg) for seven days then intoxicated with a single injection of APAP (500 mg/kg). ^*^, ^#^ significantly different from control and APAP-treated groups respectively (*p* < 0.05) using one-way ANOVA followed by the Tukey–Kramer multiple comparisons test. APAP, acetaminophen; LDH, lactate dehydrogenase

Injection of APAP significantly elevated serum LDH activity (6425.6 ± 359.3) in comparison with the control group (4377.6 ± 201.01). APAP/Nicorandil group revealed a marked reduction in serum LDH activity (5707.2 ± 261.7) in comparison with APAP-treated group (Fig. 2).

### Effect of APAP and nicorandil on hepatic antioxidant status

Injection of APAP (500 mg/kg) significantly increased hepatic MDA levels (3.6-fold change) with significant decrease in levels of GSH (by 58.1%) as well as SOD (by 15.6%) compared to the normal group. Nicorandil efficiently reversed APAP-induced alterations (reduced MDA by 62.8% besides increasing GSH by 62.1% and SOD by 16%) compared to APAP group (Table 2).

**Table 2:** Effect of APAP (500 mg/kg) and/or nicorandil (100 mg/kg) on hepatic antioxidant status in mice

	MDA (nmol/mg tissue)	GSH (nmol/mg tissue)	SOD (U/mg tissue)
Control	4.30 ± 0.05	0.1180 ± 0.002	53.50 ± 0.44
APAP	15.50 ± 1.00^*^	0.0494 ± 0.004^*^	45.14 ± 3.75^*^
APAP/Nicorandil	5.76 ± 0.54^#^	0.1304 ± 0.004^#^	53.75 ± 0.88^#^

Mice were pretreated with nicorandil (100 mg/kg) for seven days then intoxicated with a single injection of APAP (500 mg/kg)

Data expressed as means ± SEM (*n* = 10)

^*, #^ denotes significant difference at *p* < 0.05 as compared to control group and APAP-treated group respectively (one-way ANOVA and Tukey–Kramer multiple comparisons test)

*APAP*, acetaminophen; *GSH*, reduced glutathione; *MDA*, malondialdehyde; *SOD*, superoxide dismutase

### Effect of APAP and nicorandil on anti-inflammatory markers

**Fig. 3:**
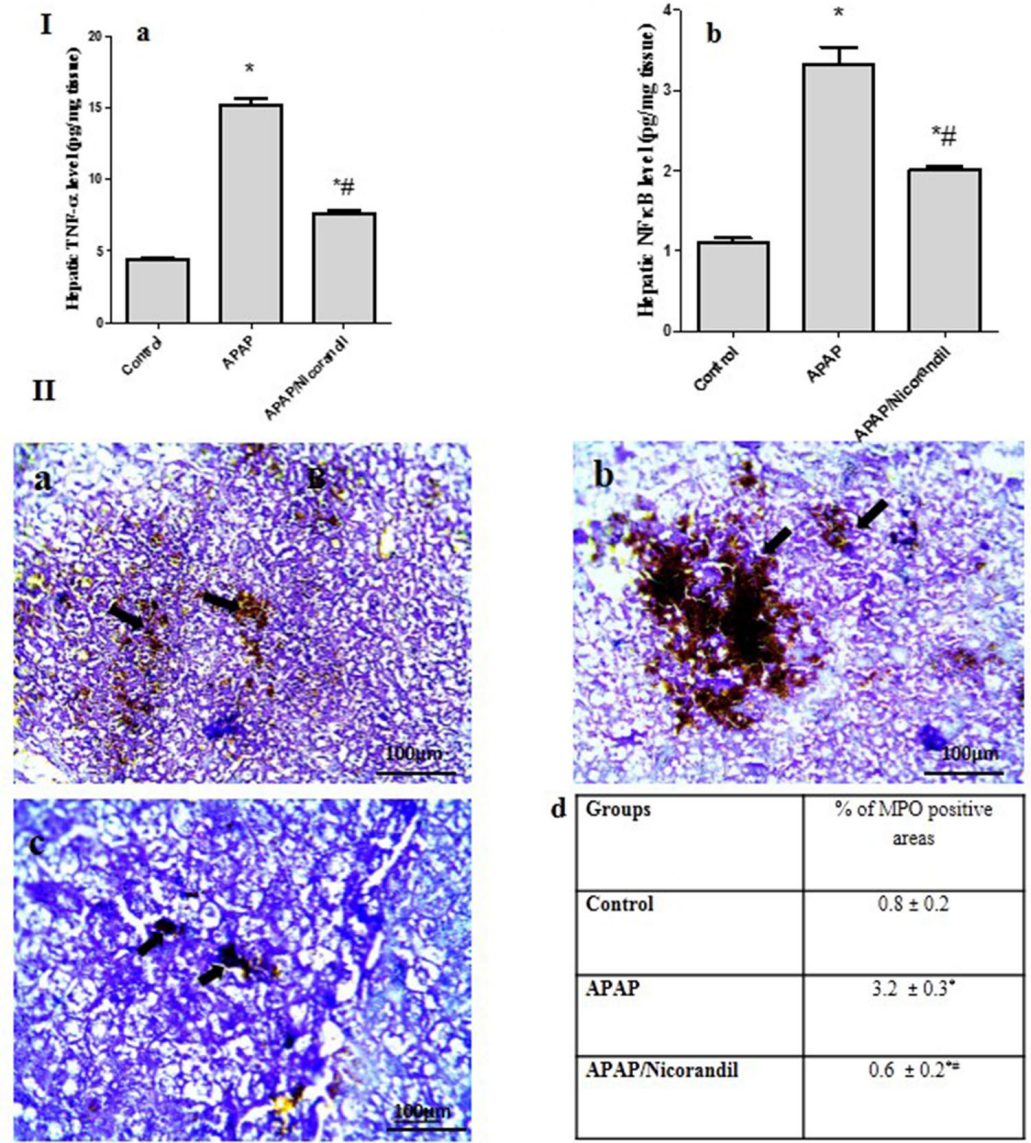
Effect of acetaminophen and nicorandil on inflammatory markers. I: Effect of acetaminophen and nicorandil on hepatic levels of TNF-α and NF-κB in mice. Mice were pretreated with nicorandil (100 mg/kg) for seven days then intoxicated with a single injection of APAP (500 mg/kg). TNF-α level (A) and hepatic NF-κB level in mice (B) were assessed. II: Effect of acetaminophen and nicorandil on MPO immunostaining A: The control group; B: The APAP group; C: The nicorandil-treated group and D: % of positive cells. Each value represents the mean of 4 mice ± S.E.M. ^*^, ^#^ significantly different from control and APAP-treated groups respectively (*p* < 0.05) using one-way ANOVA followed by the Tukey–Kramer multiple comparisons test. APAP, acetaminophen; TNF- α, tumor necrosis factor- α; NF-κB, nuclear factor- κB; MPO, myeloperoxidase

APAP-treated group produced a profound rise in hepatic TNF-α and NF-κB levels (15.2 ± 0.4 and 3.3 ± 0.2) compared to the control group (4.4 ± 0.16 and 1.1 ± 0.05). Mice treated with nicorandil revealed a significant decline in hepatic TNF-α and NF-κB levels (7.6 ± 0.2 and 2.02 ± 0.04) in comparison with APAP group (Fig. 3I A and 3I B, respectively).

Microscopic sections from hepatic tissues stained with MPO showed minimal number of stained cells in the control group (Fig. 3IIA); however, sections from APAP- group showed significant increase in number of stained cells (Fig. 3IIB). Nicorandil group exhibited mild expression of MPO (Fig. 3IIC). The immunostaining scores in nicorandil group were nearly similar to that of the control group (Fig. 3IID).

### Effect of APAP and nicorandil on antiapoptotic marker bcl-2

**Fig. 4:**
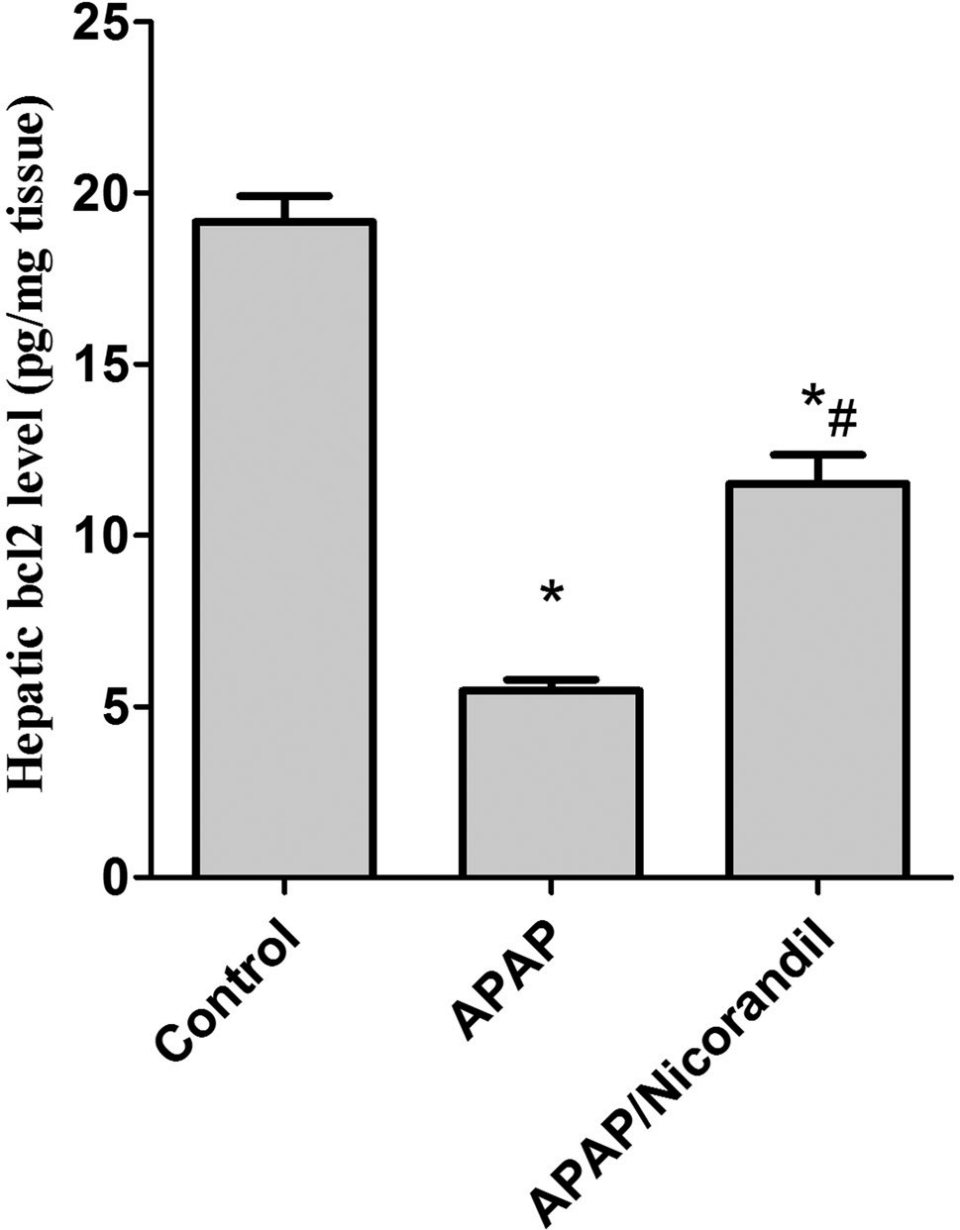
Effect of acetaminophen and nicorandil on hepatic bcl2 levels in mice. Mice were pretreated with nicorandil (100 mg/kg) for seven days then intoxicated with a single injection of APAP (500 mg/kg). Hepatic bcl2 levels were assessed. ^*^, ^#^ significantly different from control and APAP-treated groups respectively (*p* < 0.05) using one-way ANOVA followed by the Tukey–Kramer multiple comparisons test. APAP, acetaminophen; bcl-2, Cell Leukemia-2

Intoxication with APAP significantly reduced bcl-2 level (5.4 ± 0.3) compared to the control group (19.1 ± 0.7). Mice treated with nicorandil exhibited a marked increase in hepatic bcl-2 level (11.5 ± 0.8) compared to the APAP-treated group (Fig. 4).

### Effect of APAP and nicorandil on histopathological analysis

**Fig. 5:**
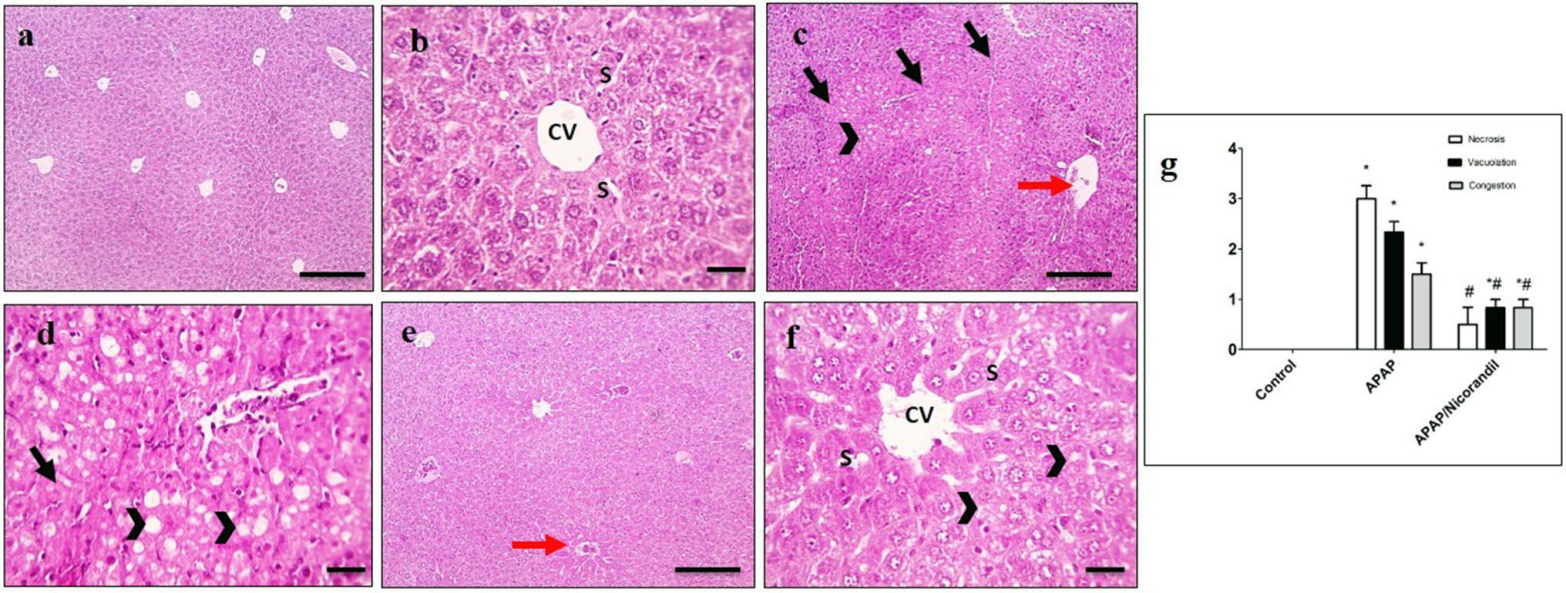
Effect of APAP and nicorandil on histopathological analysis (H&E × 100). (a & b) The control group showed normal histology of hepatic cords, central veins (CV), and sinusoids (S). (c & d) The APAP group showed confluent areas of coagulative necrosis (arrows) with large cytoplasmic vacuolation of hepatocytes (arrowheads), congestion (red arrow), and occluded sinusoids. (e & f) The nicorandil-treated group showed few minutes vacuoles of hepatocytes (arrowheads) with opened sinusoids, congestion (red arrow). a, c & e (low magnification X: 100 bar 100) and b, d & f (high magnification X: 400 bar 50). (g) Histopathological scores (necrosis, vacuolation, and congestion). ^*, #^ denotes significant difference at *p* < 0.05 as compared to control group and APAP-treated group respectively (one-way ANOVA and Tukey–Kramer multiple comparisons test) (*n* = 6). APAP, acetaminophen

Sections from control group showed normal histology of hepatic cords, central veins (CV), and sinusoids (S) (Fig. 5a, b). Liver sections from APAP-intoxicated group showed confluent areas of coagulative necrosis with large cytoplasmic vacuolation of hepatocytes, congestion, and occluded sinusoids (Fig. 5c, d). However, sections from mice treated with nicorandil showed few minutes vacuoles of hepatocytes with opened sinusoids and congestion (Fig. 5e, f). Semiquantitative scoring of hepatic lesions is shown in Fig. 5g.

### Effect of APAP and nicorandil on iNOS expression

**Fig. 6:**
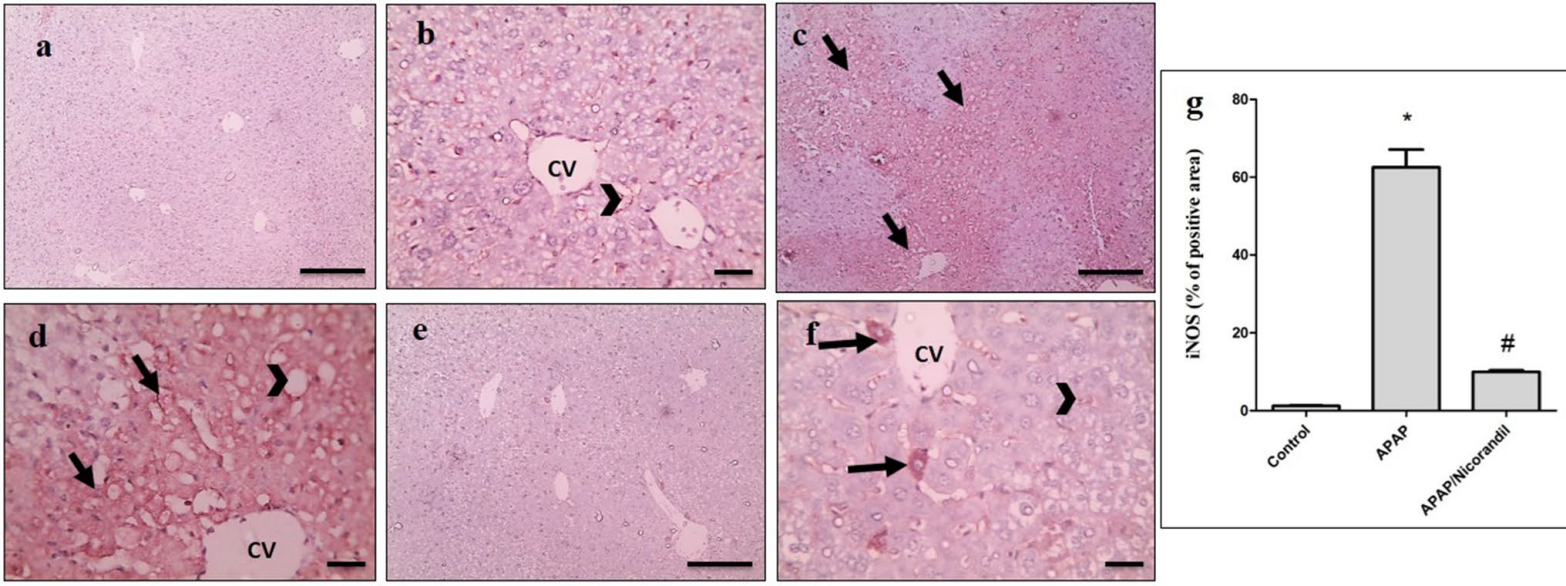
Effect of APAP and/or nicorandil on iNOS expression (IHC). (a & b) The control group showed mild positive brown staining of endothelium (arrowheads). (c & d) The APAP group showed marked marked positive brown cytoplasmic staining of hepatocytes (arrow) in areas of coagulative necrosis in addition to endothelium (arrowheads). (e & f) The nicorandil-treated group showed mild positive brown cytoplasmic staining of few hepatocytes (arrow) in addition to endothelium (arrowheads). IHC counterstained with Mayer's hematoxylin. a, c & e (low magnification X: 100 bar 100) and (b, d & f) high magnification X: 400 bar 50. (g) Percentage of iNOS immunopositive cells. ^*, #^ denotes significant difference at *p* < 0.05 as compared to control group and APAP-treated group respectively (one-way ANOVA and Tukey–Kramer multiple comparisons test) (n = 6). APAP, acetaminophen; iNOS, inducible nitric oxide synthase

Microscopic pictures of immunostained hepatic sections against iNOS from control group showed mild positive brown staining of endothelium (Fig. 6a, b). Sections from APAP group revealed marked positive brown cytoplasmic staining of hepatocytes in areas of coagulative necrosis in addition to endothelium (Fig. 6c, d). Section from nicorandil-treated group exhibited mild positive brown cytoplasmic staining of few hepatocytes in addition to endothelium (Fig. 6e, f). Percentage of positive areas is shown in Fig. 6g.

### Effect of APAP and nicorandil on eNOS expression

**Fig. 7:**
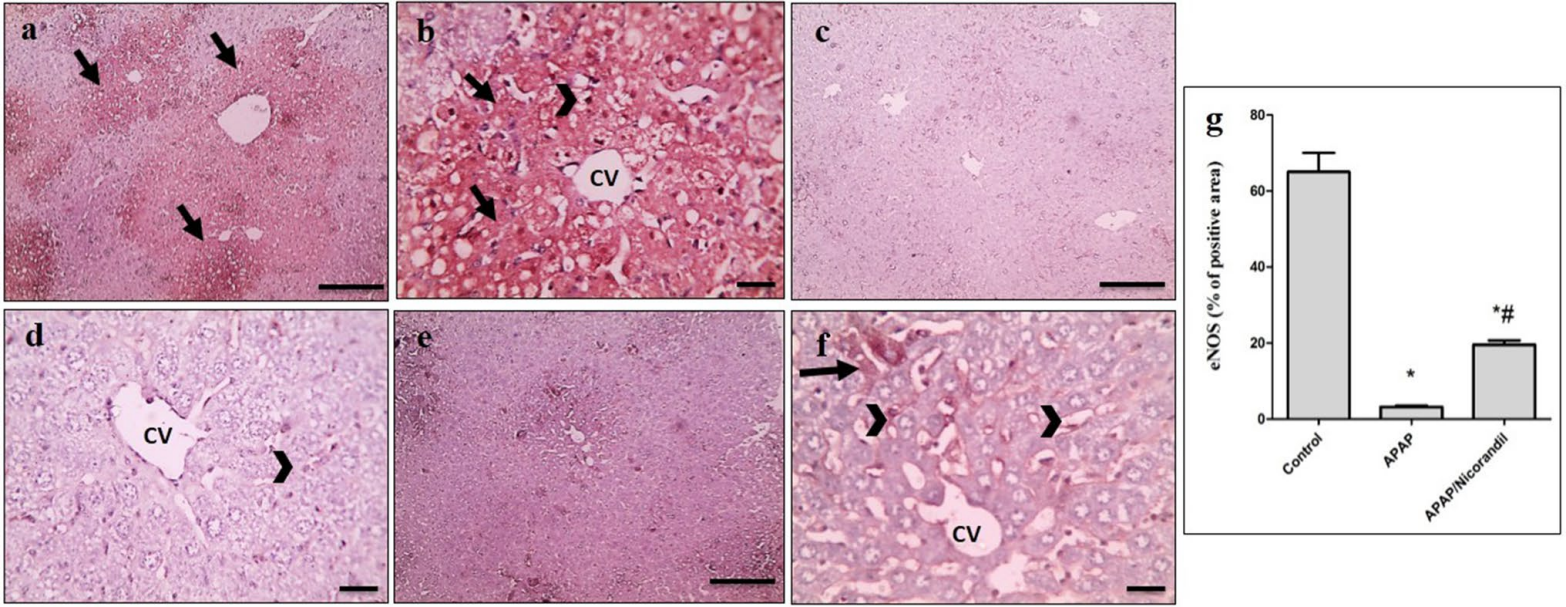
Effect of APAP and/or nicorandil on eNOS expression (IHC). (a & b) The control group showed marked positive brown cytoplasmic staining of hepatocytes (arrow) in areas of coagulative necrosis in addition to endothelium (arrowheads). (c & d) The APAP group showed mild positive brown staining of endothelium (arrowheads). (e & f) The nicorandil-treated group showed mild positive brown cytoplasmic staining of few hepatocytes (arrow) in addition to endothelium (arrowheads). IHC counterstained with Mayer's hematoxylin. a, c & e (low magnification X: 100 bar 100) and b, d & f (high magnification X: 400 bar 50). (g) Percentage of eNOS immunopositive cells. ^*, #^ denotes significant difference at *p* < 0.05 as compared to control group and APAP-treated group respectively (one-way ANOVA and Tukey–Kramer multiple comparisons test) (*n* = 6). APAP, acetaminophen; eNOS, endothelial nitric oxide synthase

Liver sections from control group showed mild positive brown staining of eNOS in endothelium (Fig. 7a, b). Conversely, sections from mice in APAP-intoxicated group showed marked positive brown cytoplasmic staining of hepatocytes in areas of coagulative necrosis in addition to endothelium (Fig. 7c, d). Treatment with nicorandil showed mild positive brown cytoplasmic staining of few hepatocytes in addition to endothelium (Fig. 7e, f). Percentage of positive areas is shown in Fig. 7g.

### Effect of APAP and nicorandil on flow cytometry (annexin V-FITC/PI)

**Fig. 8:**
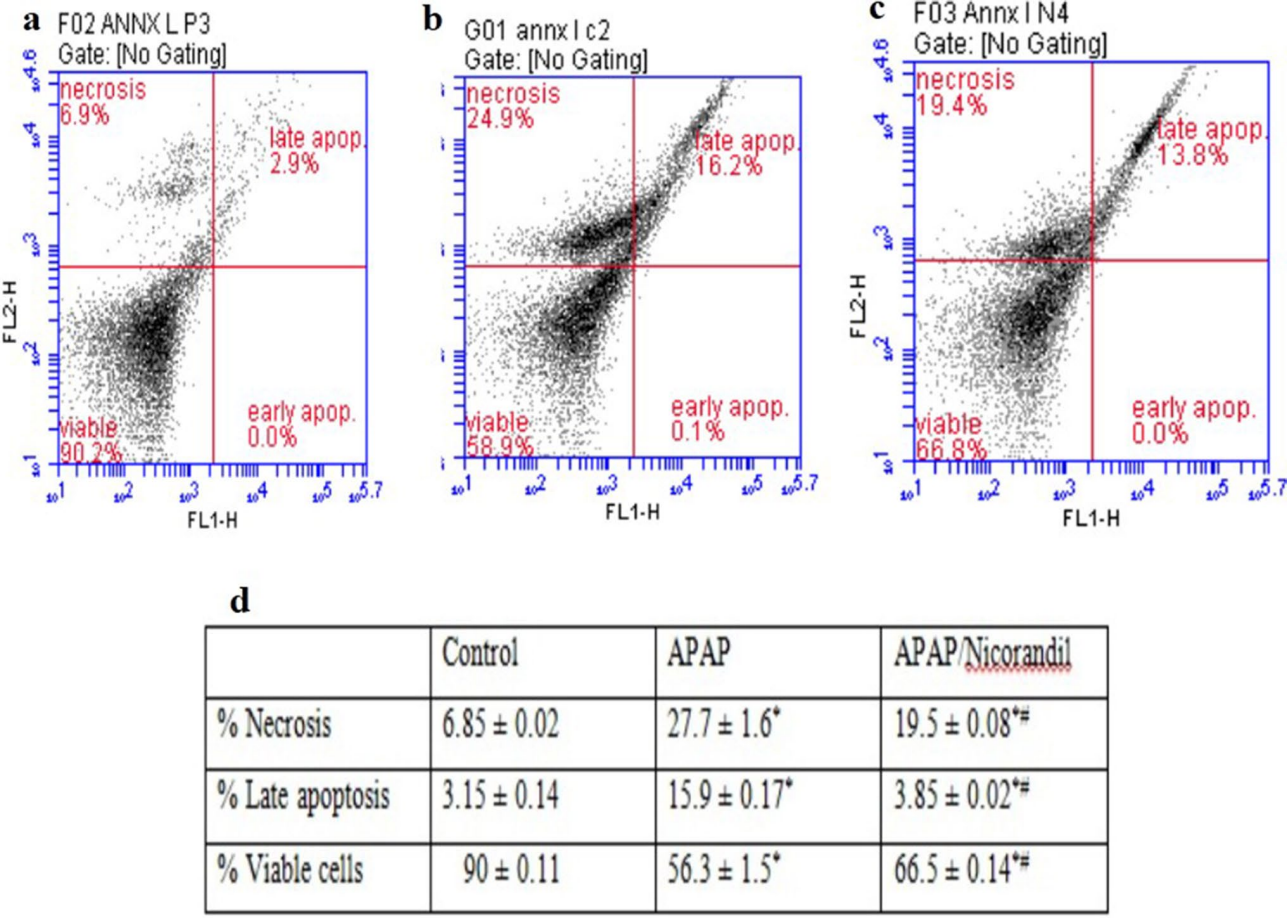
Effect of APAP and/or nicorandil on flow cytometry (annexin V-FITC/PI). Mice were pretreated with nicorandil (100 mg/kg) for seven days then intoxicated with a single injection of APAP (500 mg/kg). Apoptosis was assessed by means of flow cytometry, samples stained with annexin V-FITC and propidium iodide (PI) to evaluate cells necrosis, late apoptosis, and cell viability. (A): indicates control group chart, (B): indicates APAP group chart, (C): indicates nicorandil group chart and (D): represents a table with the percentage of viable, apoptotic, and necrotic cells. Data for flow cytometry are expressed as mean ± SEM, (*n* = 4/group). ^*^, ^#^ significantly different from control and APAP-treated groups respectively (*p* < 0.05) using one-way ANOVA followed by the Tukey–Kramer multiple comparisons test

Figure 8 clarified the effect of APAP and nicorandil on apoptosis and necrosis as well. The percent of necrotic cells and late apoptotic cells was markedly elevated, and number of viable cells were significantly reduced in APAP group (Fig. 8B) when compared to control group (Fig. 8A). Upon nicorandil administration (Fig. 8C), the percentage of necrotic cells and late apoptotic cells was markedly decreased, and the number of viable cells was significantly elevated when compared to APAP group.

## Discussion

Drug-induced liver toxicity is considered a principal cause of acute liver damage. APAP is regarded as one of the most widely reported medications that can cause acute liver damage, specifically when consumed in large toxic doses (Iorga et al. 2017). This work was designed to study the possible impacts of nicorandil on APAP–induced acute liver damage.

APAP overdose causes destruction of hepatocytes that is evidenced by the elevation in transaminase and phosphatases that cause cellular leakage and loss of functional integrity (Uchida et al. 2017). Additionally, APAP caused centrilobular hepatic necrosis as seen in histopathological examination which is in line with previous study (Hinson et al. 2010). Nicorandil ameliorated the biochemical parameters in accordance with previous studies (Elshazly 2015; Yamazaki et al. 2011). Moreover, nicorandil managed to alleviate APAP-induced necrosis seen in histopathological examination. The possibly involved mechanisms in nicorandil hepatoprotective effects were explored.

The excessive metabolism of APAP by the hepatic CYP450 results in depletion of GSH pool that increases the formation of protein adducts, causing oxidative stress that fosters systemic inflammatory response, apoptosis, and necrosis (Coen 2015; Ray et al. 1996; Xie et al. 2016). Excessive production of reactive oxygen species (ROS) causes lipid peroxidation and decrements cellular redox homeostasis (Xie et al. 2016). In this study, nicorandil showed antioxidant effect rooted on restoring GSH and scavenging free radicals in mitochondria. Increasing GSH levels by nicorandil helped in quenching NAPQI which eventually reduced the mitochondrial permeability transition (Saito et al. 2010). Additionally, nicorandil increased SOD activity and decreased MDA; these results are in line with the previously reported study of (Mano et al. 2000). The effect of nicorandil as antioxidant could be explained by its ability to inhibit free radical production through acting as a direct scavenger of hydroxyl radicals; these effects were previously reported in both human and canine leukocytes (Ozturk et al. 2017).

In liver, NO is mainly synthesized by eNOS and iNOS enzymes and it plays a crucial role in the physiology and pathophysiology of the liver (Iwakiri and Kim 2015). NO produced by iNOS could react with super oxide anion and form peroxynitrite (ONOO^¯^) which is cytotoxic to the liver in the absence of GSH (Jaeschke and Bajt 2006). Interestingly, nicorandil showed a significant decrease in NO release that could be due to diminution in iNOS protein expression. These results are in line with the reported inhibitory action of nicorandil on iNOS (Tashiro et al. 2015). Nicorandil also increased eNOS protein expression, an effect that could augment the decline in NO release. These results are in harmony with previously reported studies which showed the protective effects of nicorandil in increasing eNOS in a model of lung fibrosis and a model of acute kidney injury (Ezzat et al. 2021; Kseibati et al. 2020). It has been proposed that nicorandil elevates NO production from endothelium by activating eNOS, therefore preventing endothelial cell death. However, upregulation of iNOS, in response to inflammation, produces 100e1000-fold more NO than eNOS which has detrimental effects (Ozturk et al. 2017). So indirectly, nicorandil could inhibit the production of ONOO^¯^ and nitrotyrosine by suppressing the formation of hepatic NO and enriching SOD activity in the liver post APAP injection (James et al. 2003). These antioxidant effects of nicorandil could be also related to its action on K^+^ channels. Sato el al. reported that nicorandil cardioprotective actions were mediated by activation of ATP-sensitive K^+^ channels in the mitochondria (mitoK_ATP_) (Sato et al. 2000). Previous notion showed that MitoK_ATP_ is the same type that is present in primary hepatocytes and thus using a selective MitoK_ATP_ opener can enhance liver regeneration after partial hepatectomy. Additionally, MitoK_ATP_ openers were proved to reverse oxidative stress and cellular damage found in case of ischemia (Nakagawa et al. 2012; Ramírez et al. 2016). Furthermore, a study reported that activating MitoK_ATP_ channels could suppress inflammatory cytokines production which could be attributed to inhibition of mitochondrial ROS production. This effect offers cardiac protection in a model of ischemia/reperfusion (Ebrahimi et al. 2014).

In APAP-induced liver damage, infiltrating neutrophils are considered a potent source of oxidative stress. They produce superoxide anion by NADPH oxidase resulting in the formation of hydrogen peroxide. MPO, which is considered a marker of inflammation and neutrophil infiltration, uses the produced hydrogen peroxide to generate potent oxidant hypochlorite which can cause direct cytotoxicity (Abdelrahman and Abdel-Rahman 2019; Adams et al. 2010; Du et al. 2016). In APAP model, oxidative stress triggers many intracellular signaling pathways resulting in the increase in pro-inflammatory cytokine production contributing to the pathogenesis of acute liver damage.

Elevation in LDH combined with elevation of ALT are considered markers of hepatocyte injury (Kotoh et al. 2008). Moreover, the major transcriptional factor NF-κB regulates the pro-inflammatory pathway; it stimulates cytotoxic factors such as iNOS and pro-inflammatory cytokines such as TNF-α, which is a prominent initiator in APAP-induced liver damage (Schwabe and Brenner 2006; Xie et al. 2016). Our results showed that APAP significantly increased levels of LDH, NF-κB and TNF-α, and the protein expression of MPO in hepatic tissues. These findings are supported by earlier studies which reported that APAP induced inflammation in hepatic tissues (Xie et al. 2016; Zhao et al. 2020). However, pre-treatment with nicorandil significantly decreased these inflammatory markers supporting our notion that nicorandil has anti-inflammatory properties; these observations agreed with previously reported studies (Ahmed and El-Maraghy 2013; El-Kashef 2018b; Elshazly 2015).

Apoptosis has a critical role in APAP-induced hepatocytes toxicity (Kon et al. 2007). In our study, annexin V/PI staining showed an increase in late apoptotic and necrotic cells in APAP group. These findings are in tune with previously reported data on the effect of APAP on human hepatoma cells and lymphocytes which suggests that APAP-induced cell death can be caused primarily by apoptosis, late stages of apoptosis, and subsequently secondary necrosis (Boulares et al. 2002). Furthermore, the anti-apoptotic protein bcl-2 negatively controls the mitochondrial release of pro-apoptotic proteins. Also, it has been associated not only with apoptosis but also with programmed form of necrotic death (Nikoletopoulou et al. 2013). The anti-apoptotic effect of nicorandil is exhibited in the present study; it thrived to lower the number of late apoptotic cells (shown by annexin V/PI stain) and to increase viable cells, besides its ability to elevate bcl-2 protein level. These findings agreed with the previous literature of Nishikawa et al. (Nishikawa et al. 2006). Correspondingly, nicorandil inhibitory action against apoptosis has been reported to be mediated via activation of mitoK_ATP_. This effect can suppress nuclear breakdown and inhibit apoptotic events thus maintaining the integrity of mitochondria and cellular functions (Nagata et al. 2003). Thus, nicorandil effects on repressing mitochondrial apoptotic signaling cascade might be due to its dual mechanism through opening of mitoK_ATP_ and/or through its nitrate-like effect (Sasaki et al. 2000).

## Conclusion

Nicorandil could be a putative prophylactic drug against APAP-induced acute liver damage. Nicorandil regulates NO homoeostasis and possesses antioxidant effect that causes reduction in inflammatory and apoptotic responses. The curative treated effect of nicorandil against APAP-induced liver injury has not been investigated in this study, which is considered a limitation for this research. Additionally, further in vitro studies are needed to estimate the inhibitory dose 50 (IC_50_) of nicorandil on NOS. Similarly, clinical studies will be needed to approve the use of nicorandil as a hepatoprotective agent.

## Data Availability

The datasets generated and/or analyzed during the current study are not publicly available (we do not have the permission to share) but are available from the corresponding author on reasonable request.
